# Editorial: Diversity Oriented Synthesis

**DOI:** 10.3389/fchem.2018.00668

**Published:** 2019-01-24

**Authors:** Andrea Basso, Seung Bum Park, Lisa Moni

**Affiliations:** ^1^Dipartimento di Chimica e Chimica Industriale, Università degli Studi di Genova, Genova, Italy; ^2^Department of Chemistry, CRI Center for Chemical Proteomics, Seoul National University, Seoul, South Korea

**Keywords:** diversity orientated synthesis, multicomponent reactions (MCRs), fragment based drug design, chemoimformatics, principal component analysis—PCA, heterocycles

Small molecules play an essential role in the field of drug discovery and chemical biology. It is therefore fundamental to have synthetic methodologies capable of assembling molecules characterized by innovative molecular frameworks, both in terms of skeleton and of stereochemistry. Diversity-oriented synthesis (DOS) aims to explore, through rapid and efficient synthetic methodologies, unexplored areas of the biology-relevant chemical space, to find new bioactive molecules, possibly aimed toward new biological targets (Burke and Schreiber, 2004; Galloway et al., 2010).

One of the most successful approaches applied in DOS is the build/couple/pair approach. This approach has been used efficiently in the past by numerous research groups, and several success stories. Yi et al. in their short review illustrate how the build/couple/pair approach can be applied to the synthesis of natural product-like compounds and macrocycles. Since this thematic issue looks more to the future than to the past, Yi also illustrates a more recent strategy, ring-distortion, which aims to achieve molecular diversity by altering pre-existing cyclic systems, through ring closures or openings, ring expansions or contractions, and other transformations. Through this approach the synthesis of natural product-like molecules, as well as macrocycles and benzannulated compounds, can be achieved.

Within this context, Kidd et al. demonstrated that the typical and well-tested build/couple/pair approach is currently used to produce DOS libraries as part of the fragment-based drug discovery (FBDD) (Erlanson and Jahnke, 2016), as opposed to the past, where DOS libraries were almost exclusively analyzed through HT screenings. Particularly interesting are those cases where new synthetic methodologies are introduced in the DOS strategy, as for example the C–H activation or the site-selective late-stage modifications of complex scaffolds. In fact, the use of these methodologies meets one of the requirements necessary for such molecules to be efficiently exploited in the FBDD, which is also one of the stated objectives of DOS, i.e., to *escape from flatland* (Lovering et al., 2009) and therefore to generate 3-dimensionally diversified molecules with a globular structure and abundance of chiral centers and sp3 carbons.

Other ways to assemble 3-dimensionally complex structures were presented by Lenci et al., who exploited morpholine skeletons rich in sp3 carbon atoms, derived from sugars and amino acids, and by Moni et al., who exploited spiro-fused ring systems as scaffolds for the generation of three DOS libraries. Both groups benefitted from chemoinformatic analysis of chemical space, to gain insight into the detailed structure of the molecular scaffolds and to verify the validity of synthetic strategy. Specifically, calculation of the Principal Component Analysis was used by Lenci and Trabocchi to group the synthetized molecules into four main clusters, depending on both their main skeletons and side-chain properties.

The work by Moni et al. reveals, among other things, how multicomponent reactions, combining three or more diversomers in a single step, are useful to assemble DOS libraries in a straightforward manner. Along the same lines is the report by Maskrey et al. which, exploiting one of the oldest multicomponent reactions, the Biginelli reaction, manages to obtain fused polyheterocycles, through a cascade process involving a hetero Diels-Alder reaction, in a stereoselective fashion.

The possibility to use the multicomponent Ugi reaction, followed by post-condensation cyclizations, to obtain polyheterocyclic systems, was analyzed in detail by Bariwal et al., who explored the various synthetic routes of assembling small/medium-sized rings, taking examples from recent literature.

A comprehensive review was also published by Murlykina et al. who, taking advantage of their expertise on the properties and reactivity of amino-azole derivatives, illustrate the strategies that can lead to DOS libraries from these compounds, both by exploiting two-component reactions as well as multicomponent processes. The synthesis of nitrogen-containing polyheterocycles, useful both in the field of drug-like substances and functional materials, is the subject of the article by Wu et al. The authors show how the DOS approach can be used to generate imidazothiazole-based chemical probes capable of identifying the biological targets implied in epigenetic processes.

As was the case for combinatorial chemistry, which was initially developed in the field of drug discovery, but then found wide application in other fields, such as materials science, the same phenomenon is happening for DOS, and Grotkopp et al. demonstrated this with their studies on luminescent materials. The DOS approach developed herein enables a rapid assembly of dual emissive bichromophores, to be employed for accessing unimolecular white light emitters for OLED and biophysical analytics.

In conclusion, going back to the question made at the beginning of this thematic issue, i.e., whether the diversity-oriented synthesis approach is still valid or is experiencing a contraction, we can now answer that the concept of DOS is still validly applicable, and improvements and deviations from its original guidelines are taking DOS in new directions, already showing very interesting results in various research fields.

## Author Contributions

All authors listed have made a substantial, direct and intellectual contribution to the work, and approved it for publication.

### Conflict of Interest Statement

The authors declare that the research was conducted in the absence of any commercial or financial relationships that could be construed as a potential conflict of interest.
